# Gastroprotective Effect of Zingerone on Ethanol-Induced Gastric Ulcers in Rats

**DOI:** 10.3390/medicina55030064

**Published:** 2019-03-11

**Authors:** Neda Sistani Karampour, Ardeshir Arzi, Anahita Rezaie, Marzieh Pashmforoosh, Fatemeh Kordi

**Affiliations:** 1 Department of Pharmacology, Jundishapur University of Medical Sciences, Ahvaz 6135715794, Iran; n.sistani1386@yahoo.com; 2 Department of Pathobiology, Faculty of Veterinary Medicine, Shahid Chamran University of Ahvaz, Ahvaz 6135783151, Iran; a.rezaie@scu.ac.ir; 3 Department of Pharmacology, Jundishapur University of Medical Sciences, Ahvaz 6135715794, Iran; marzie_pf@yahoo.com (M.P.); f.pharmacy_89@yahoo.com (F.K.)

**Keywords:** gastric ulcer, zingerone, ethanol, malondialdehyde, nitric oxide, rat

## Abstract

*Background and objectives:* Zingerone is an ingredient of ginger (*Zingiber officinale*) with different pharmacological activities. Several studies have investigated the effect of zingerone on various gastrointestinal diseases, including irritable bowel syndrome and diarrhea. This study is aimed to evaluate the effect of zingerone on ethanol-induced gastric ulcers in rats. *Materials and Methods:* Gastric ulcers were induced by ethanol (96%, 5 mL/kg, po) in male wistar rats and zingerone (50, 100, and 200 mg/kg) was administrated orally. Normal saline and ranitidine were used as negative and positive control, respectively. In this study, the number and length of ulcers, and malondialdehyde (MDA) and nitric oxide (NO) levels in stomach tissues were determined. *Results:* The findings showed that the mean number and length of gastric ulcers were significantly lower in zingerone-received groups than ethanol group (*P* < 0.05). The level of malondialdehyde was decreased in the stomach of zingerone groups (*P* < 0.05) compared to the ethanol group. In addition, zingerone treatment prevented the decrease of nitric oxide level by ethanol in the stomach tissue. *Conclusions:* The present study showed that zingerone has a protective effect on the ethanol-induced gastric ulcer, which may be due to its free radical scavenging activity.

## 1. Introduction

The peptic ulcer has been considered as one of the most common digestive diseases in the present century [1]. Peptic ulcers are chronic and often single lesions that may occur in any part of the digestive tract [2]. In the United States, the risk of developing peptic ulcer disease is 10% over a lifetime. The pathophysiology of this disease has a multifactorial process that is caused by the imbalance between aggressive factors, in particular, acid and pepsin on one hand, and mucosal defense factors, especially blood flow and prostaglandins, on the other [3]. Factors that may increase the incidence of peptic ulcer disease (PUD) include stress, alcohol consumption, smoking, *Helicobacter pylori*, and the use of nonsteroidal anti-inflammatory drugs (NSAIDs) [4].

Although using antibiotics, proton pump inhibitors (omeprazole), prostaglandin analogs, and H2 receptor blockers (cimetidine, ranitidine, and famotidine) reduce the mortality of stomach ulcers, attempts to discover new drugs with lower cost and fewer side effects are necessary [5].

Natural products are an important source for the prevention and treatment of gastric ulcer [6]. Various studies have demonstrated that ginger (*Zingiber officinale*) possesses gastroprotective effects in experimental gastric ulcer models [7,8,9]. Zingerone (4 (4-hydroxy-3-methoxyphenyl)-2-butanone) is a non-toxic ingredient of ginger (*Zingiber officinale*) with different pharmacological activities. It has strong anti-inflammatory, anticancer, anti-diabetic, anti-diarrheal, antispasmodic, anti-thrombotic, and antimicrobial properties [10]. The antioxidant activity of zingerone has been shown in various experimental animal models of diseases and it is shown that this effect of zingerone is equal to ascorbic acid [11,12].

Several studies have been conducted on the effect of zingerone on various gastrointestinal diseases. Among these studies, treatment of irritable bowel syndrome through the effect on intestinal smooth muscle [13], treatment of vomiting induced by chemotherapy [14], diarrhea [15], and colitis [16] is remarkable. However, the gastroprotective effect of zingerone has not been studied. 

Based on the potent anti-inflammatory and antioxidant effects of zingerone, it seems that zingerone may possess the beneficial therapeutic effect on the gastric ulcer produced by ethanol. Therefore, this study is aimed to evaluate the effect of zingerone on ethanol-induced peptic ulcers in rats.

## 2. Materials and Methods

### 2.1. Chemicals

Zingerone was purchased from Sigma Aldrich (St. Louis, MO, USA) and ethanol (96%) was purchased from Khorramshahr Co. (Khorramshahr, Iran).

### 2.2. Animals

Male wistar rats weighing 200–250 g were purchased from the animal house of the Ahvaz Jundishapur University of medical sciences (Ahvaz-Iran). To prevent coprophagia, the animals were placed in single cages with raised mesh bottoms and kept under standardized conditions of temperature (22 ± 1 °C). Feeding was withdrawn 24 h prior to the experiment, however rats had access to water ad libitum. The study was approved by the Institutional Ethical Committee of the School of Medicine, Ahvaz Jundishapur University of Medical Sciences guidelines for animal care and use (IR.AJUMS.REC.1395.642).

### 2.3. Induction of Acute Gastric Lesion by Ethanol

Gastric ulcer was induced by administration of 96% ethanol (5 mL/kg, po). One hour after ethanol instillation, the animals were anesthetized with intraperitoneal administration of ketamine (50 mg/kg) and xylazine (10 mg/kg). The stomachs were dissected and opened along greater curvature for evaluating the number and the length of gastric lesions [17]. A portion of the stomach was dissected and was subsequently homogenized in cold potassium phosphate buffer (0.05 M, pH 7.4). The homogenate was centrifuged at 5000 rpm for 10 min. The supernatant was stored at −80 °C until measurement of malondialdehyde and nitric oxide [18].

### 2.4. Experimental Design

The animals were randomly divided into the following groups: vehicle group (normal saline + normal saline), the ethanol group (normal saline + ethanol), treatment group (zingerone + ethanol), the positive control group (ranitidine + ethanol), and a control group received zingerone (200 mg/kg) without ethanol administration [19]. The treatment groups were orally received zingerone at doses of 50, 100, and 200 mg/kg. The route of administration and doses of zingerone were based on previous studies [20,21]. Six animals in each group were used for the experiments.

### 2.5. Determination of Malondialdehyde (MDA) in Gastric Mucosa

The level of lipid peroxidation was determined as malondialdehyde according to the commercial instructions of the colorimetrical assay kit (ZellBio GmbH, Ulm, Germany) by measurement of thiobarbituric acid (TBA)-reactive the substance at 535 nm.

### 2.6. Determination of Nitric Oxide (NO) in Gastric Mucosa

The content of nitric oxide in gastric mucosa was measured according to the commercial instructions of the colorimetrical assay kit (ZellBio GmbH, Ulm, Germany). The nitric oxide level was assessed quantitatively based on its metabolites (nitrite/nitrate) and Griess reaction.

### 2.7. Histopathological Examination

For histopathology assessment, stomach tissues were fixed in 10% buffered formalin solution and were embedded in paraffin. Sections were deparaffinized and stained with hematoxylin and eosin (H&E).

### 2.8. Statistical Analysis

Data were expressed as mean ± S.E.M. Comparisons between means of different groups were analyzed by one-way analysis of variance (ANOVA) followed by Tukey’s post hoc. The level of significance was taken as *P* < 0.05. The Graph Pad Prism software package, version 5 (Graph Pad Software, Inc., San Diego, CA, USA), was used to carry out all statistical tests.

## 3. Results

### 3.1. Effect of Zingerone on Ethanol-Induced Gastric Lesions

**Figure 1:**
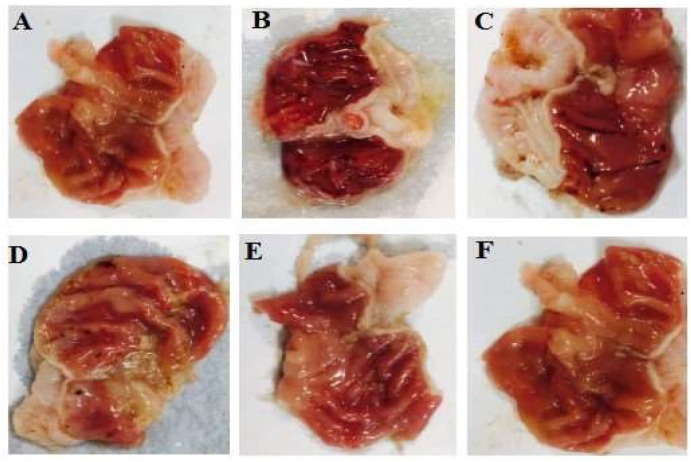
The gross appearance of gastric ulcers induced by ethanol (5 mL/kg) and protective effects of zingerone (50, 100, or 200 mg/kg). Normal saline + normal saline (**A**), normal saline + ethanol (**B**), ethanol + zingerone (50 mg/kg) (**C**), ethanol + zingerone (100 mg/kg) (**D**), ethanol + zingerone (200 mg/kg) (**E**), and ethanol + ranitidine (50 mg/kg) (**F**). Ethanol caused extensive hemorrhagic ulcers (B) and treatment with zingerone at doses of 100 and 200 mg/kg attenuated gastric ulcers.

**Figure 2:**
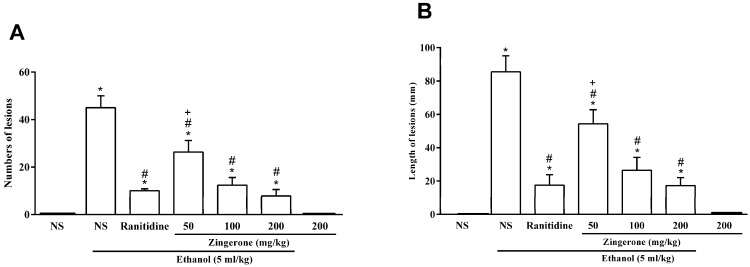
The effect of zingerone (50, 100, or 200 mg/kg) on the (**A**) numbers and (**B**) length of gastric ulcers (in mm) induced by ethanol. Values are expressed as mean ± S.E.M. (*n* = 6). * *P* < 0.05 compared to the normal saline (NS). ^#^ *P* < 0.05 compared to the normal saline and ethanol. ^+^ *P* < 0.05 compared to the ranitidine.

As shown in Figure 1, the ethanol (96%) administration induced extensive long and hemorrhagic gastric ulcers in the rat. Oral pretreatment with zingerone attenuated the number and the length of gastric lesions in a dose-depended manner (*P* < 0.05, Figure 2A,B). Among the tested doses, high doses of zingerone (200 mg/kg) showed maximum inhibition on the number and length of gastric lesions. Ranitidine (50 mg/kg) and zingerone (100, 200 mg/kg) had similar effects on the number and length of gastric ulcers (Figure 2). There was no significant difference between the gastroprotective activity of dose 100 mg/kg and dose 200 mg/kg zingerone. In addition, high doses of zingerone (200 mg/kg) alone had no side effects on stomach tissue.

### 3.2. Effect of Zingerone on Malondialdehyde (MDA) Level in Gastric Tissue

**Figure 3:**
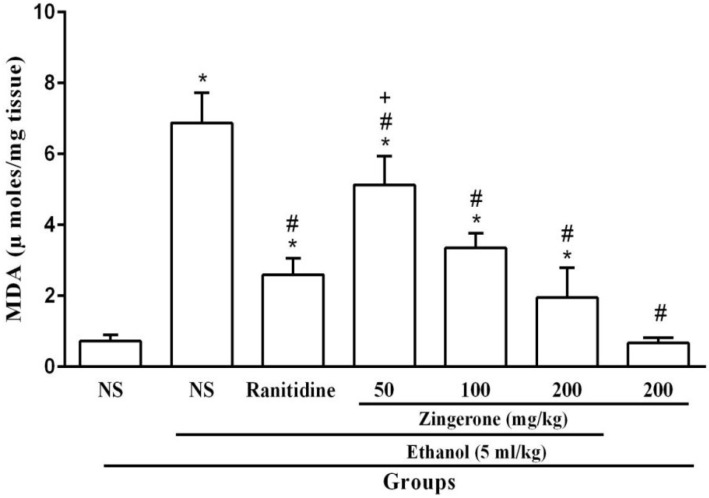
Effect of zingerone (50, 100, or 200 mg/kg) on the levels of malondialdehyde (MDA) in gastric tissues. Values are expressed as mean ± S.E.M.; (*n* = 6). * *P* < 0.05 compared to the normal saline (NS). ^#^ *P* < 0.05 compared to the normal saline and ethanol. ^+^ *P* < 0.05 compared to the ranitidine.

As shown in Figure 3, ethanol increased the gastric MDA level compared to the normal saline group (*P* < 0.05, Figure 3). Administration of zingerone at the doses of 100 and 200 mg/kg, similar to the ranitidine group, exhibited a significant reduction in MDA level in stomach tissue (*P* < 0.05).

### 3.3. Effect of Zingerone on Nitric Oxide (NO) Level in Gastric Tissue

**Figure 4:**
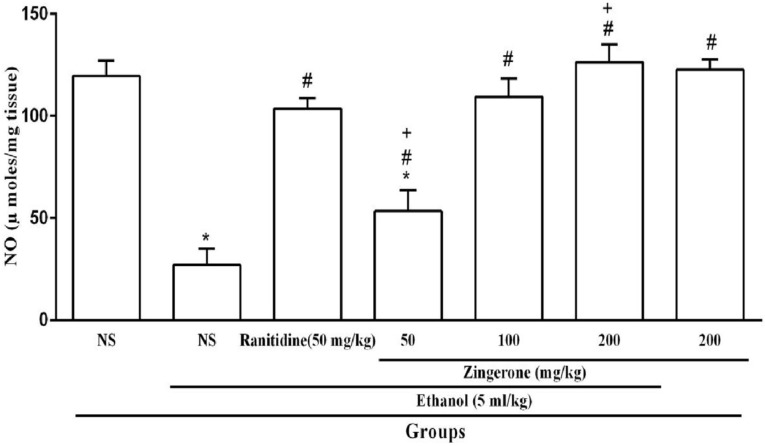
Effect of zingerone (50, 100, or 200 mg/kg) on the levels of nitric oxide (NO) in gastric tissues. Values are expressed as mean ± S.E.M.; (*n* = 6). * *P* < 0.05 compared to the normal saline (NS). ^#^ *P* < 0.05 compared to the normal saline and ethanol. ^+^ *P* < 0.05 compared to the ranitidine.

The results (Figure 4) showed that ethanol administration significantly decreased the gastric nitric oxide level in rats. Administration of zingerone (100 or 200 mg/kg) restored the nitric oxide level in the stomachs exposed to ethanol (*P* < 0.05, Figure 4).

### 3.4. Histopathology

**Figure 5:**
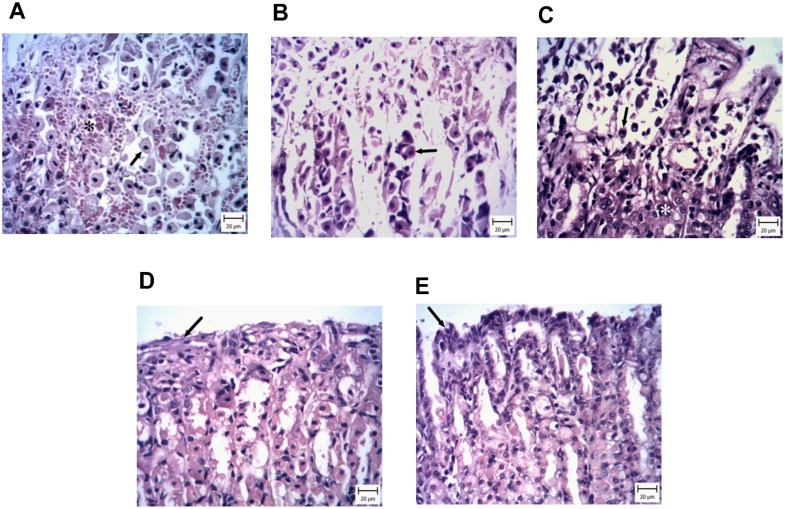
Protective effects of zingerone (50, 100, or 200 mg/kg) on histopathological changes of gastric tissue induced by ethanol (hematoxylin and eosin staining, magnification ×100). (**A**) Normal saline and ethanol, (**B**) ethanol and pretreatment with 50 mg/kg zingerone, (**C**) ethanol and pretreatment with 100 mg/kg zingerone, (**D**) ethanol and pretreatment with 200 mg/kg zingerone, (**E**) ethanol and ranitidine (50 mg/kg). The results showed (A): Note to hemorrhage (asterisk), epithelial cell necrosis (arrows), and desquamation of them which are indicators of gastric ulcer, (B): Massive area of hemorrhage and necrosis of the epithelium (arrows), (C): Note to necrotic cells (arrow) and normal cell (white asterisk), (D): Note to normal desquamation of epithelial cells (arrow) and normal glandular cells, (E): Note to normal epithelial cells (arrow) and normal glandular cells.

Histopathological evaluation of the stomach tissue sections showed that the ulcer size and the number of ulcers in the ethanol group were greater than other groups and severe necrosis was seen in the epithelial and gland cells. These cells had dark nuclei with eosinophilic cytoplasm and they were detached from the lower layer. In this area, hemorrhages were obvious (Figure 5A). In treatment groups, ulcers were seen, but they were decreased at doses of 100 mg and 200 mg/kg of zingerone. In the 50 mg/kg group, the ulcers were greater than the ethanol group, but the severity was the same (Figure 5B). In the 100 mg/kg group, the ulcers were confined to the upper part of the glands (Figure 5C). In the 200 mg/kg group, the number of ulcers decreased, and the mucosal cells showed necrosis, but glands were normal (Figure 5D). In addition, the control group showed the normal structure (Figure 5E).

## 4. Discussion

Despite many advances in the therapeutic management of gastric ulcers, the prevalence of this disease is still high [22]. Many phytochemical studies have shown that the phenolic compounds possess an important role in the prevention of gastric ulcer [23]. For the first time, this study demonstrates that administration of zingerone at the doses of 100 and 200 mg/kg decreases the lipid peroxidation in the stomach and attenuates the ethanol-induced gastric ulcer in the rat.

Ethanol-induced gastric ulcer is a common animal model to investigate the new anti-ulcer drugs [24]. Administration of ethanol causes gastric necrotic damage and subsequent inflammatory cell infiltration and reduces the secretion of bicarbonate, gastric mucus, and nitric oxide. In addition, ethanol reduces the gastric blood flow and induces the oxidative stress by increasing the production of malondialdehyde and reducing glutathione production [25]. The present study showed that zingerone at doses 50, 100, or 200 mg/kg reduced the histopathologic changes and the number and size of gastric ulcers induced by ethanol in the rat. It was also found that zingerone at doses of 100 and 200 mg/kg had an equal effect with ranitidine on the number and size of gastric ulcers. Various studies have shown the anti-ulcerogenic effect of ginger. Wang et. al reported the gastroprotective effect of ginger in aspirin-induced gastric ulcer model rat [9]. Minaiyan et. al showed the anti-ulcerogenic effect of ginger on the cysteamine-induced duodenal ulcer in rat [26]. Our study suggests that part of the gastroprotective effect of ginger is due to zingerone.

It is well-known that oxidative stress and reactive oxygen species (ROS) are involved in the pathogenesis of ethanol-induced gastric ulcer. The gastric ulcer induced by ethanol is associated with the increased purine degradation that leads to increased O2^-^ radical production and ROS-mediated increased lipid peroxidation. [27]. Several studies have shown that scavenging free radicals by antioxidant compounds prevent ethanol-induced gastric ulcer [28,29]. In agreement with previous studies, the present finding showed that ethanol administration in rats led to a significant increase in MDA level in stomach tissue [29,30]. Pretreatment with zingerone (100 and 200 mg/kg) could decrease the level of malondialdehyde in the gastric tissue. Our data are consistent with various reports that have shown that zingerone is a potent free radical scavenger in various tissues, including liver, heart, and kidney [10]. Banjie et al. found that zingerone has a direct effect on intestinal muscle, which leads to healing the damage caused by irritable bowel syndrome because of the nature of energization as well as the strong antioxidant nature [13]. Rao et al. observed that zingerone neutralizes free radicals induced by radiation [31]. Moreover, Hemalata et al. showed that zingerone protects the rat’s heart against isoproterenol-induced myocardial infarction due to its free radical scavenging activity [32]. Reducing the level of malondialdehyde as the major marker of lipid peroxidation in rat stomach can be a gastroprotective effect of zingerone due to eliminating the free radicals and reducing oxidative stress. It was proposed that the free radical scavenging activity of zingerone might be related to the methoxy group and a long-chain ethyl methyl ketone group in its chemical structure [33]. Therefore, it seems that at least part of the protective effect of zingerone on ethanol-induced gastric ulcers may be due to its potent antioxidant activity and ability to scavenging free radicals.

Various reports showed that nitric oxide plays a protective role in gastric ulcer, and treatment with NO donors can accelerate the healing of gastric ulcer [34,35]. It was indicated that the protective effects of nitric oxide in gastric ulcer are related to the gastric mucosal blood flow, mucus secretion, and inhibition of inflammation [36]. In this study, to determine the role of nitric oxide in the gastroprotective effect of zingerone in the ethanol-induced gastric ulcer, gastric NO level was measured. The current study demonstrated that ethanol administration decreased the gastric level of nitric oxide. These findings are in agreement with previous studies [28,34]. Zingerone (100 and 200 mg/kg) treatment could inhibit the reduction of the gastric nitric oxide level by ethanol. However, the previous report showed that zingerone could increase the production of NO and guanylate cyclase activity in rat aorta [35], but in this study, there was not any significant difference in NO level between zingerone-received groups and the control group. The mean nitric oxide levels in the receiving groups of 100 mg/kg and 200 mg/kg zingerone were significantly higher than those receiving ranitidine. The gastroprotective effect of zingerone might be related to the nitric oxide pathway.

## 5. Conclusions

According to our results, zingerone exerts anti-ulcerogenic effect and can be effective in reducing the incidence of gastric ulcer and its complications. However, more studies are needed to determine the exact mechanisms of antiulcer activity of zingerone.
